# DD-CC-II: Data Driven Cell–Cell Interaction Inference and Its Application to COVID-19

**DOI:** 10.3390/ijms262010170

**Published:** 2025-10-19

**Authors:** Heewon Park, Satoru Miyano

**Affiliations:** 1 School of Mathematics Statistics and Data Science, Sungshin Women’s University, Seoul 01133, Republic of Korea; 2 M&D Data Science Center, Institute of Science Tokyo, Tokyo 113-8510, Japan; 3 Human Genome Center, Institute of Medical Science, University of Tokyo, 4-6-1 Shirokane-dai, Minato-ku, Tokyo 108-0071, Japan

**Keywords:** cell–cell interactions, eigen-cell, over-representation analysis, COVID-19 severity stage, disease progression of COVID-19

## Abstract

Cell–cell interactions play a pivotal role in maintaining tissue homeostasis and driving disease progression. Conventional Cell–cell interactions modeling approaches depend on ligand–receptor databases, which often fail to capture context-specific or newly emerging signaling mechanisms. To address this limitation, we propose a data-driven computational framework, data-driven cell–cell interaction inference (DD-CC-II), which employs a graph-based model using eigen-cells to represent cell groups. DD-CC-II uses eigen-cells (i.e., functional module within the cell population) to characterize cell groups and construct correlation coefficient networks to model between-group associations. Correlation coefficient networks between eigen-cells are constructed, and their statistical significance is evaluated via over-representation analysis and hypergeometric testing. Monte Carlo simulations demonstrate that DD-CC-II achieves superior performance in inferring CCIs compared with ligand–receptor-based methods. The application to whole-blood RNA-seq data from the Japan COVID-19 Task Force revealed severity stage-specific interaction patterns. Markers such as FOS, CXCL8, and HLA-A were associated with high severity, whereas IL1B, CD3D, and CCL5 were related to low severity. The systemic lupus erythematosus pathway emerged as a potential immune mechanism underlying disease severity. Overall, DD-CC-II provides a data-centric approach for mapping the cellular communication landscape, facilitating a better understanding of disease progression at the intercellular level.

## 1. Introduction

Cell–cell interactions (CCIs) are essential for tissue homeostasis and driving disease progression. Understanding cell communication within complex biological systems is key to unravelling multicellular organization and function. Recent strategies to model CCIs based on possible ligand–receptor pairs include a computational model to calculate CCI likelihood [1]. Various computational methods have been developed to infer cell–cell interactions (CCIs) based on ligand–receptor (L–R) co-expression patterns. Sum-based and correlation-based approaches, such as CellCall and REMI, quantify communication strength using L–R expression levels adjusted by transcription factor activity or correlation coefficients [2,3]. Differential expression-based methods, e.g., iTALK, identify significantly regulated ligands and receptors to interpret intercellular communication [4]. Product- or permutation-based models, including CellChat, NATMI, and scSeqComm, estimate interaction probabilities using geometric or statistical normalization strategies [5,6,7]. More recent machine learning frameworks, such as CellGDnG and CellDialog, leverage ensemble or deep learning techniques to enhance L–R interaction prediction [8,9]. In addition, CellPhoneDB computes enrichment-based interaction scores by averaging L–R expression within annotated cell types [10]. Collectively, these methods have been widely applied to explore disease progression, immune regulation, and tissue development by modeling intercellular communication networks.

However, existing methods rely heavily on static ligand–receptor databases, which may not fully capture context-specific interactions or newly discovered signaling mechanisms. Data-driven computational frameworks are being utilized to address these challenges and predict potential intercellular communication. Specifically, graph-based approaches representing cells as nodes and potential interactions as edges have emerged as powerful tools for reconstructing cell communication landscapes.

This study proposes a novel computational strategy, DD-CC-II, for data-driven CCI interference using a graph-based approach. The associations and dependencies between subjects are represented as a network of nodes (cell groups) connected by edges (links). The strength of these associations is measured by the correlation coefficient network of eigen-cells. The significance of the association between the cell groups can be assessed using an overrepresentation analysis of eigen-cell pairs.

The performance of DD-CC-II was assessed in the current study using Monte Carlo simulations and whole-blood RNA-seq data from the Japan COVID-19 Task Force [11], which analyses COVID-19 samples according to severity stages (asymptomatic, mild, severe and critical) as cell groups. The interaction between COVID-19 stages was inferred based on the eigen-cells of each severity stage. The results revealed pivotal genes involved in the early stages of COVID-19 that are significantly implicated in progression to more severe stages. These markers were validated through a comprehensive literature survey. The findings suggest that targeting severity-specific markers could help prevent the progression to more severe stages of COVID-19. Our proposed DD-CC-II strategy is a fully data-driven, graph-based framework for inferring cell–cell interactions. Unlike conventional methods that rely on static ligand–receptor databases, our approach characterizes cell groups using SVD-derived eigen-cells, models inter-group association through an eigen-cell correlation network, and rigorously evaluates significance using over-representation analysis and the hypergeometric test. This enables the robust, system-level inference of cell–cell communication, representing a significant methodological advance over existing tools, thereby enhancing our understanding of tissue organization, immune regulation, and disease pathology at the cellular network level.

A limitation of our approach is that only cancer types with at least two sublines containing sufficient numbers of cells were included to ensure robust statistical inference. Consequently, the generalizability of our strategy to rare cancers may be limited, as their cellular heterogeneity and interactions might not be fully captured in the current dataset.

## 2. Results

### 2.1. Monte Carlo Simulation 1: DepMap Dataset

A Monte Carlo simulation was conducted to assess the performance of DD-CC-II based on generated synthetic datasets with the ground truth known. Statistical evaluation results were provided through repeated sampling, ensuring that performance metrics were not artifacts of a specific dataset.

We used a publicly available CCLE expression dataset from the DepMap database (https://depmap.org/portal/ (accessed on 1 February 2020)), comprising 19,221 genes and 1406 cells from more than 20 types of cancer (i.e., lineages). The lineage subtypes of each cancer were defined as distinct cell groups, selecting only cancer types with diverse sub-lineages and a sufficient number of cells. Specifically, sub-lineages comprising more than ten cells were considered individual cell groups; only cancer types containing more than two sub-lineages were included in the analysis. Consequently, CCI inferences were made for cells from lung, blood, lymphoid tissue, breast, soft tissue, and bone cancers. The cell groups for each cancer type are presented in Table 1. Previous studies demonstrated enhanced ligand–receptor communication within the same lineage subpopulations in various cancer types, including glioma, lung adenocarcinoma, and colorectal cancer [12,13,14]. Tang et al. [12] revealed numerous significant ligand–receptor interactions among neoplastic cells, including those associated with autocrine and paracrine signaling within the same tumor lineage. Meanwhile, Yang et al. [13] demonstrated that tumor cell sub-lineages within the same lung adenocarcinoma lineage engage in direct communication via shared ligand–receptor expression. Similarly, Lin et al. [14] defined active ligand–receptor interactions between different subpopulations of malignant epithelial cells, indicating robust communication among sub-lineages within the same colorectal cancer lineage.

In the current study, the interactions between cell groups (i.e., sub-lineages) in the same lineage were considered true positives of the CCI inference. For instance, interactions between groups of cells in non-small cell lung cancer (NSCLC), small-cell lung cancer (SCLC), and mesothelioma were considered true positives of CCI inference for lung cancer. The eigen-cells of a specific sub-lineage were estimated using the expression levels of cells in the sub-lineage and a randomly selected 5% of cells for other lineages. The eigen-cells that did not belong to a sub-lineage were estimated by using SVD based on the expression levels of randomly selected cells for other lineages. That is, eigen-cells for NSCLC, SCLC, and mesothelioma (EGs-NSCLC, EGs-SCL, and EGs-mes) were estimated based on 141 cells (135 NSCLC cells + 5% of 135 non-lung cells), 52 (50 SCLC cells + 5% of 50 non-lung cells) and 21 (20 mesothelioma cells + 5% of 20 non-lung cells), respectively. For false positives, eigen-cells not involved in lung cancer (EGs-nLC1, EGs-nLC2, and EGs-nLC3) were estimated by randomly selecting 141, 52, and 21 cells of lineages other than lung cancer. The interactions among EGs-nLC1, EGs-nLC2, and EGs-nLC3 were considered CCI false positives.

The selection of singular values was generally guided by the criterion that their cumulative variance contribution exceeds a threshold within the 70–90% range [15,16]. In line with previous research, we selected the number of eigen-cells ($Q_{g}$) based on the number of singular values required to capture 75% of the cumulative variance in the expression profiles of each sub-lineage. Table 2 presents the numbers of eigen-cells (i.e., $Q_{g}$) capturing 75% variance of cell groups, where EGs-*x* and EGs-n*x* indicate the cell groups for cancer sub-lineages (e.g., EGs-1: NSCLC, EGs-2: SCLC, EGs-3: mesothelioma in lung cancer) and randomly generated groups of cells, respectively.

**Figure 1:**
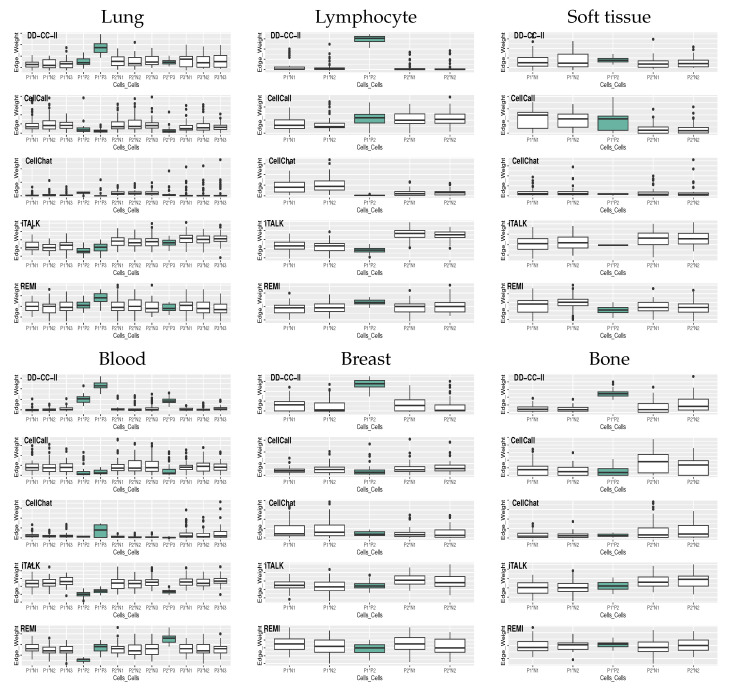
DeepMap dataset: Strength of the association between cell groups, where P*x* and N*x* indicate the true positive and true negative of the *x*th cell group, respectively. Interactions of type Px*P*x* correspond to true positives, whereas Nx*P*x* and Nx*N*x* correspond to false positive associations.

Eigen-cell correlation networks between EGs-NSCLC, EGs-SCL, EGs-mes, EGs-nLC1, EGs-nLC2, and EGs-nLC3 constructed using significant correlation coefficients with a significant level α=0.05 (i.e., *p*-value ≤α). The significance of associations between groups was determined using the hypergeometric test. Similar procedures were applied to infer the CCI of other cancers (blood, lymphocyte, breast, soft tissue, and bone). The proposed strategy was evaluated by comparing it with existing strategies for CCI analysis: CellCall, CellChat, iTALK, and REMI. Using existing methods, the strength of the association (i.e., edge weight) was computed between cell groups. In CellCall, CCI strength is measured as the ligand–receptor score between groups based on intercellular signaling (ligand and receptor expression levels) and intracellular signaling (activity of downstream transcription factors). CellChat computes the edge weight based on total communication probability of ligand–receptor pairs between groups. The edge weights of CCIs in iTALK represent the average expression levels of the ligand gene in a given cell type (i.e., value of “*cell_from_mean_exprs* ” in the *iTALK package version 0.1.0* of *R*). In REMI, the strength of the association between cell groups is expressed as the number of ligand–receptor interactions detected between cell types. Detailed descriptions of the existing methods for CCI inference have been provided elsewhere by [2,3,4,5]. We measured the significance of the association based on FDR-q. values and described the edge weights as −log(FDR-q.value). The simulation was performed over 50 iterations. Figure 1 shows the strength of the association between groups of cells computed by CellCall, CellChat, iTALK, REMI, and DD-CC-II in the 50 simulations, where the green boxes indicate the true positives of CCIs. DD-CC-II appropriately achieved CCI inference, as evidenced by the designation of relatively larger edge weights for true positive interactions between cell groups (i.e., Px*P*x*) than for false positive interactions (i.e., Nx*P*x*, Nx*N*x*). In contrast, existing methods (i.e., CellCall, CellChat, iTALK, and REMI) failed to effectively perform CCI inference; the strength of the association was not described accurately.

**Figure 2:**
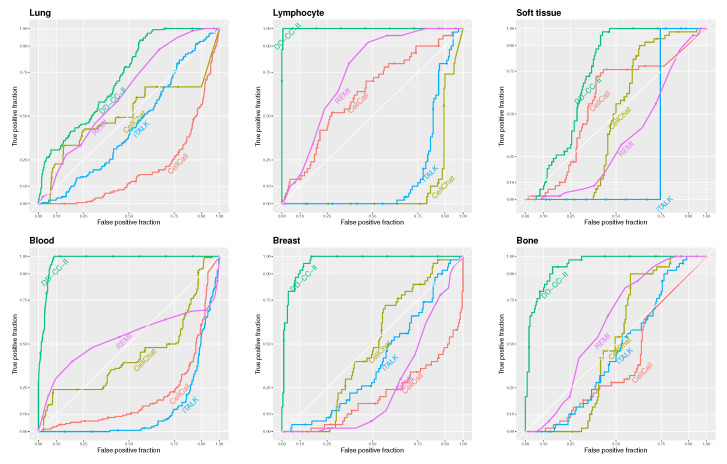
Receiver operator characteristic (ROC) curves for DD-CC-II, CellCall, iTALK, and REMI based on the cut-off values of the edge strength.

Figure 2 shows the receiver operator characteristic (ROC) curves for DD-CC-II, CellCall, CellChat, iTALK, and REMI based on the threshold of the edge weights described in Figure 2. Our strategy outperformed other methods in CCI inference. In particular, DD-CC-II exhibited an outstanding performance for CCI inference of lung, lymphocyte, blood, breast, and bone cancers. In contrast, the other methods, particularly iTALK, performed poorly.

We evaluated the methods for inferring CCIs based on the area under the curve (AUC) of the ROC curves. Given that our scenarios involve a small number of true CCIs relative to all possible pairings (i.e., a class imbalance), we additionally assessed performance using the AUC of the precision–recall (PR) curves, which are more appropriate for imbalanced datasets. Table 3 reports the AUC values of both ROC and PR curves; values in parentheses indicate the standard deviations of AUC scores across 50 simulation runs. To assess the sensitivity of the results of DD-CC-II to significant level α for correlation coefficient network, we also described the results (AUC values) based on α=0.01 in Table 3, where columns α=0.05 and α=0.01 indicate AUC values of DD-CC-II based on the correlation coefficient network with α=0.05 and 0.01, respectively.

The results also show that our strategy will be a useful tool for CCI inference and evaluating cell signaling. Furthermore, Table 3 also demonstrates that using a threshold of α=0.05 in the construction of the correlation coefficient network yields better results than using α=0.01. This result suggests that the performance of DD-CC-II is sensitive to the threshold used in constructing the correlation coefficient network. Therefore, selecting an appropriate threshold is a critical issue that warrants careful consideration, and the application of multiple testing correction should be taken into account. However, given that DD-CC-II demonstrated more effective results with a relatively higher threshold (i.e., α=0.05) compared to α=0.01, multiple testing correction was not applied in this analysis.

### 2.2. Monte Carlo Simulation 2: GDSC Database

DD-CC-II was also applied to the “Sanger Genomics of Drug Sensitivity in Cancer (GDSC) dataset from the Cancer Genome Project”. The gene expression data (Cell_line_RMA_proc_basalExp.txt) comprised 9764 genes in 968 cells from more than 30 cancer types. Cell groups were generated based on primary tissue type classification (i.e., GDSC Tissue descriptor 1) related to more than two cancer types with more than ten cells, i.e., aero_dig_tract (AERO), leukaemia (LEUL), digestive_system (DIG), nervous_system (NERV). Table 4 lists the cell groups for each cancer type.

Similar to the analysis of the DeepMap dataset, the interactions between cell groups (i.e., cancer types) in the same lineage/primary tissue type classification were considered true positives of the CCI inference. That is, the eigen-cells of a specific cancer type were estimated using the expression levels of cells in the cancer type and 5% randomly selected cells for other cancer types. The eigen-cells that did not belong to a cancer type were also estimated based on the expression levels of randomly selected cells for other categories of primary tissue type classification (i.e., true negative scenario). The number of eigen-cells (e.g., $Q_{g}$) was set to the number of singular values capturing 75% variance in the expression levels of each sub-lineage, as shown in Table 5. The eigen-cell correlation networks were constructed with a significant level α=0.05. The CCI inference based on correlation coefficient networks were performed similar to the CCI inference of DeepMap data.

**Figure 3:**
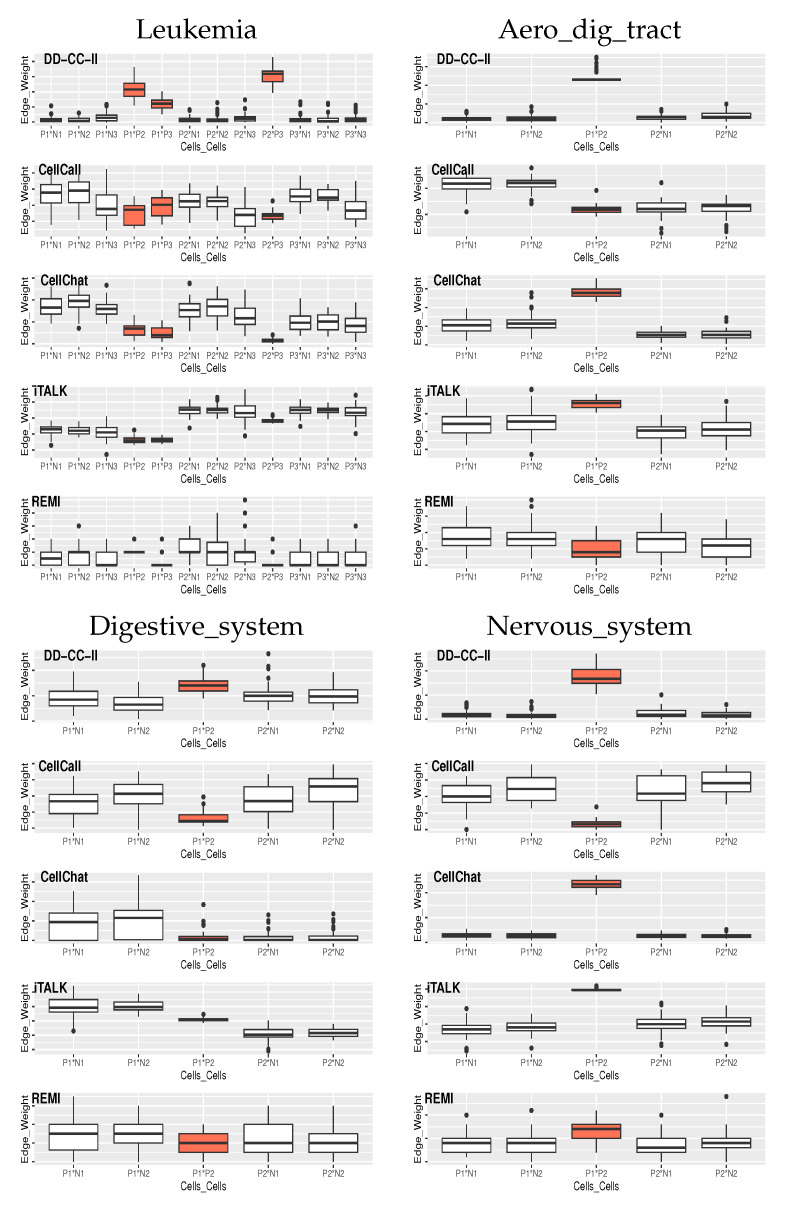
GDSC dataset: Strength of the associations between cell groups, where P*x* and N*x* indicate the true positive and negative of the *x*th cell group, respectively.

Figure 3 shows the edge weights between the groups of cells estimated by the CellCall, CellChat, iTALK, REMI, and DD-CC-II in the 50 simulations, where the orange boxes indicate the true positives of CCIs.

DD-CC-II also exhibited an outstanding performance for CCI inference, i.e., edge weight estimation in CCIs (Figure 3). Although the CellChat and iTALK also appropriately achieved CCI inference for Aero_dig_tract and Nervous_system, they did not generate effective CCI results for Leukemia and Digestive_system.

Table 6 shows the AUC values of the ROC and PR curves, where the numbers in parentheses correspond to the standard deviation of the AUC values obtained from 50 simulations. Consistent with the results of the DeepMap dataset, the proposed DD-CC-II shows outstanding performances for the CCI inference of cancer types.

Finally, CCI inference in terms of computational complexity was evaluated, where the CCI execution times were assessed based on DD-CC-II, CellCall, CellChat, iTALK, and REMI for CCIs for DepMap and GDSC datasets using the R package (see Table 7). The proposed DD-CC-II demonstrated competitive performance in terms of computational complexity compared to the existing methods. In contrast, CellCall demonstrated a considerable computational burden.

### 2.3. Uncovering Disease Trajectory Correlations Between COVID-19 Severity Stages

COVID-19 is a severe infectious disease, particularly for those with critical illnesses who are at high risk of rapid deterioration. Dinsay et al. [17] reported in-hospital mortality rates of 5.4%, 8.1%, 27.0%, and 80.3%, for mild, moderate, severe, and critical COVID-19 cases in the Philippines, respectively. Meanwhile, in Turkey, mortality rates were 4.7% for mild-to-moderate cases, 23.9% for severe cases, and 100% for critical cases [18]. Preventing COVID-19 progression is crucial for better clinical outcomes. Accordingly, we sought to characterize correlations between COVID-19 severity stages and key markers involved in disease progression. We considered samples from each severity stage as a cell group and measured the strength of the association between the COVID-19 severity stages based on the eigen-cell links for each stage. DD-CC-II was applied to the whole blood RNA-seq data of 1102 genotyped samples provided by the Japan COVID-19 Task Force; the COVID severity stages were defined as “critical (Level 4: patients in intensive care unit or requiring intubation and ventilation),” “severe (Level 3: others requiring oxygen support),” “mild (Level 2: other symptomatic patients),” and “asymptomatic (Level 1: without COVID-19 related symptoms)” [11]. The RNA-seq expression data of COVID-19 samples are available at the National Bioscience Database Center (NBDC) Human Database (accession code: hum0343; https://humandbs.biosciencedbc.jp/en/hum0343, (accessed on 1 April 2022)).

The RNA-seq data for 71 asymptomatic, 241 mild, 404 severe, and 303 critical samples were considered as four groups of cells and applied to construct eigen-cell correlation networks. Particular focus was placed on genes involved in the “*Coronavirus disease-COVID-19*” pathway, i.e., COVID-19 genes in the KEGG pathway database. Subsequently, disease-trajectory correlations between COVID-19 stages were inferred based on the eigen-cells of each stage computed by the expression levels of the COVID-19 genes. To elucidate the mechanisms associated with immune damage in COVID-19, DD-CC-II was also applied to disease-trajectory correlations between COVID-19 severity stages based on the genes involved in “*immune disease*” pathways, i.e., immune disease-related genes. Table 8 presents the KEGG database “*Coronavirus disease-COVID-19*” and “*immune disease*” pathways. For the genes involved in each pathway, eigen-cells were estimated for samples corresponding to each COVID-19 stage and used to construct eigen-cell correlation coefficient networks. Finally, DD-CC-II was applied to identify disease-trajectory correlations for COVID-19 severity stages. For each pathway, we examined whether severe COVID-19 groups showed significant associations. To control the false positive rate from multiple comparisons, Bonferroni correction was applied, and associations with an FDR-q.value less than 0.05 were considered significant. All genes were included in the eigen-cell construction. Lowly expressed genes were not excluded; their contributions to the eigenvectors are naturally weighted according to their expression levels, allowing all genes to influence the representation while reducing the dominance of highly variable genes. Table 9 lists the FDR-q.value of the disease-trajectory correlations analysis. As shown in Table 9, the severity stages of COVID-19 computed by the COVID-19 genes show a relatively strong association with those computed by immune disease-related genes.

**Figure 4:**
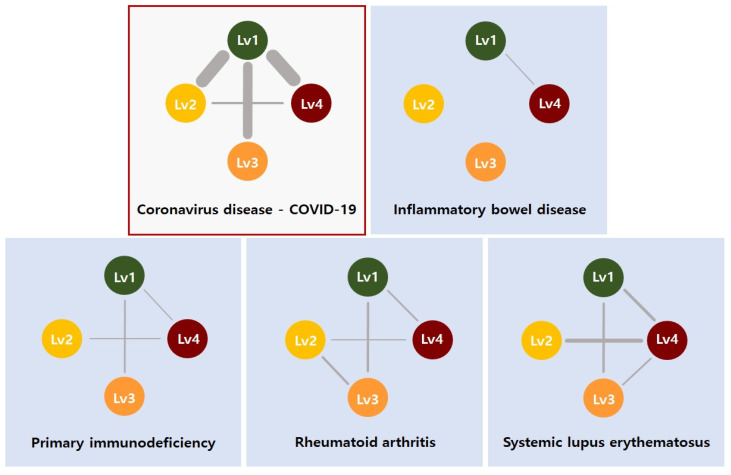
Interaction between COVID-19 severity stages for genes involved in *Coronavirus disease-COVID-19* and *Immune disease* pathways.

Figure 4 (upper right) presents the disease-trajectory correlations between COVID-19 severity stages based on COVID-19 genes. In COVID-19 severity stage interactions, asymptomatic samples (Level 1) were strongly associated with mild (Level 2), severe (Level 3), and critical (Level 4) samples. This implies that mild, severe, and critical stages of COVID-19 may have similar gene transcription patterns as asymptomatic samples. Hence, genes with key roles in the initial stages of COVID-19 may also be critical for later-stage disease progression. Table 10 presents the crucial genes in eigen-cell estimation for each COVID-19 severity stage, where rank indicates the ranking of the absolute loading values for the first eigen-cell estimation. The highly ranked genes can be considered crucial markers for understanding COVID-19 mechanism. The crucial genes for the eigen-cell estimation of the initial stages (asymptomatic samples; Level 1), that is, HLAB, HLAC, NFKBIA, RPS11, RPS27, and RPL41, were also identified in the eigen-cell estimation for higher stages (mild: Level 2, severe: Level 3, critical: Level 4 samples). These common genes have been suggested previously as COVID-19 markers (Table 10).

- HLA
  Naidoo et al. [19] reported that HLAB mRNA expression affects COVID-19 severity and links to ethnic differences in susceptibility. In particular, HLA class I alleles may be critical in determining COVID-19 severity [20]. Weiner et al. [21] proposed that HLA class I alleles play a significant role in immune defence against COVID-19. Genetic variations in HLA help regulate immune responses to COVID-19, contributing to individual differences in infection susceptibility and severity [22]. Zhang et al. [23] reported HLAB allelic expression patterns and overexpression in SARS-CoV-2-infected human lung epithelial cells.
- Ribosome Protein (RPS and RPL family)
  Persistent viral infection in COVID-19 patients may be associated with immunosuppression and decreased ribosome protein expression [24].
- NFKBIA
  Reduced NFKBIA mRNA stability can diminish the ability of IκBα to retain NF-κB in the cytoplasm [25]. Indeed, NF-κB gene variants may contribute to the likelihood of developing severe COVID-19. Moreover, NF-κB-related genes, including TNFAIP3, NFKBIA, and FOS, become upregulated in epithelial cell lines 8 h post-SARS-CoV-2 infection [26].

In contrast, FOS, CXCL8, and HLA-A were revealed as high-severity-specific markers that were identified not in asymptomatic patients but in mild, severe, and critical patients.

- FOS
  High FOS expression is a key feature of COVID-19 patients [27], making it a potentially promising target for managing SARS-CoV-2 infection [28,29]. Similarly, Lu et al. [30] observed a strong association between FOS and nonalcoholic steatohepatitis and COVID-19.
- CXCL8
  Elevated CXCL8 levels have also been reported in early COVID-19 patients’ blood and alveolar spaces [31], with higher levels in severe cases but no significant increase in mild cases compared to healthy controls [32]. Hence, downregulated inflammatory marker genes, particularly CXCL8, may serve as powerful biomarkers for managing COVID-19 infection [33]. According to Park and Lee [32], HLAA also significantly influences COVID-19 severity across ethnicities.

Collectively, these results suggest that suppressing high severity-specific markers (i.e., FOS, CXCL8, and HLA-A) may help prevent COVID-19 progression.

The disease-trajectory correlations between COVID-19 stages computed by immune-related genes are also presented in Figure 4. The COVID-19 stages show relatively weak associations with the immune-related genes compared with the COVID-19 genes. Moreover, “*inflammatory bowel disease*”, “*primary immunodeficiency*”, “*Rheumatoid arthritis*”, and “*systemic lupus erythematosus*” were identified as immune damage pathways underlying COVID-19, with significant COVID-19 stage interactions estimated based on genes involved in these four pathways. The association between mild and critical samples were common for immune-related pathways. Additionally, the COVID-19 severity stage cells for genes involved in the “*Systemic lupus erythematosus*” pathway exhibited relatively strong association and active interplay (i.e., numerous edges). This implies that the “*Systemic lupus erythematosus*” pathway is crucial in defining the mechanism and progression of COVID-19 stages. The “*Systemic lupus erythematosus*” pathway was highlighted due to its relevance to immune dysregulation in COVID-19, including aberrant type I interferon signaling [34]. Shared molecular mechanisms and severity-specific enrichment suggest its role in modulating immune responses during disease progression [35]. The “*Immune disease*” genes that are crucial for eigen-cell estimation are listed in Table 11. Similarly to eigen-cell estimation based on the COVID-19 genes, many common genes were identified as crucial markers. IL1B, CD3D, CD4, CCL5, and SNRPB were identified as low-severity-specific markers.

- IL1β
  The elevated levels of intestinal IL-1β have been linked to the longer survival and lower levels of intestinal SARS-CoV-2 [36]. Moreover, patients with severe COVID-19 and poor prognosis have lower levels of IL1B, IL2, and IL8 compared to those with favorable outcomes [37]. Hence, IL-1β could serve as a key marker for targeted treatment in patients with COVID-19 [38,39].
- CD3D
  CD3D was also identified as a core gene linked to immune infiltration, with potential diagnostic utility in COVID-19 patients with sepsis. Zhang et al. [40] proposed that a risk score based on CD3D, CD3E, LCK, and EVL could serve as a predictive model for severe COVID-19.
- CD4
  CD4+ T cells are significantly diminished in severe COVID-19 cases [40]. Meanwhile, CD4-mediated SARS-CoV-2 infection of T helper cells can contribute to a weakened immune response in patients with COVID-19 [41]. However, SARS-CoV-2-specific, TNF-α-producing CD4+ T cells are crucial in maintaining antibody titres after COVID-19 infection [42].
- CCL5
  CCL5 has been described as the optimal indicator of COVID-19 severity [43]. CCL5 levels negatively correlate with mortality in COVID-19, suggesting that it may protect against severe disease progression [44]. In particular, CCL5 is significantly upregulated from the early stages of infection in those with mild disease, but not in severe cases [45]. Thus, enhancing CCL5 expression early in COVID-19 may reduce the risk of severe illness. Therefore, monitoring CCL5 levels could predict infection severity [46] and be applied to inform treatment strategies [45].

JAK3, ICAM1, and H2BC4 were identified as markers of higher COVID-19 severity. These results are strongly supported by those of existing research, implying that our strategy provides biologically reliable results for the disease-trajectory correlations of COVID-19 stages and related marker identification. The role of SNRPB in COVID-19 has not yet been explored, indicating that it could be considered a novel potential biomarker for the disease.

- JAK3
  Sbruzzi et al. [47] identified a novel homozygous JAK3 variant in a patient with severe COVID-19, suggesting that JAK3 may represent a key marker for persistent infection. In patients with cirrhosis, elevated plasma ICAM1 acts as an independent predictor of severe COVID-19 [48].
- ICAM1
  ICAM1 serves as a prognostic marker for long-term complications or sequelae due to COVID-19 infection [49] and an effective biomarker for predicting COVID-19 severity [50].

**Figure 5:**
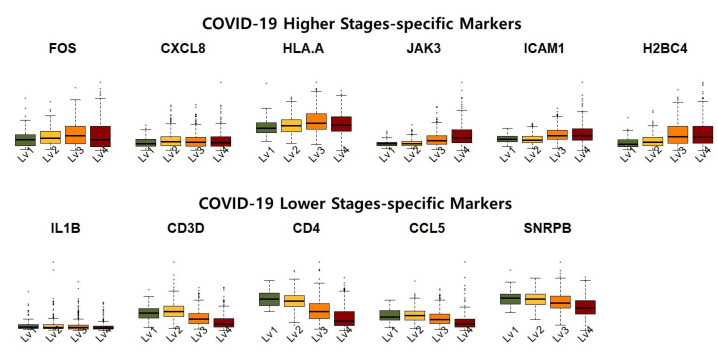
Expression of COVID-19 low severity and high severity-specific markers in asymptomatic (Lv1), mild (Lv2), severe (Lv3), and critical (Lv4) samples.

Figure 5 shows the expression levels of the identified COVID-19 high-severity and low-severity-specific markers. The COVID-19 high-stage-specific markers (i.e., FOS, CXCL8, HLAA, JAK3, ICAM1, and H2BC4) were overexpressed in higher-stage samples, that is, increased expression levels of the markers were observed in asymptomatic to critical samples. In contrast, the low-stage-specific markers (i.e., IL1B, CD3D, CD4, CCL5, SNRPB) were relatively upregulated in the lower-stage samples. Furthermore, the expression of high (low)-stage-specific markers exhibited considerable variance in severe (non-severe) samples. This result implies that high (low)-stage-specific markers exhibited high transcriptional activity in samples of COVID-19 high(low) stages.

Based on our results, we suggest that controlling high-severity-specific markers (FOS, CXCL8, HLA-A, JAK3, ICAM1, and H2BC4) and low-severity-specific markers (IL1B, CD3D, CD4, CCL5, and SNRPB) may prevent COVID-19 progression. We also suggest that the “*Systemic lupus erythematosus*” pathway is crucial to understanding the mechanisms underlying COVID-19 stage progression.

## 3. Discussion

In this study, a novel data-driven strategy for CCI inference, DD-CC-II, was developed. This approach characterizes cell groups using eigen-cells and subsequently constructs eigen-cell correlation networks. To evaluate the significance of the association between cell groups, an over-representation analysis was conducted on the correlation networks.

Monte Carlo simulations were performed to demonstrate the performance of the proposed DD-CC-II. The simulation results demonstrated that our data-driven framework achieved superior performance in inferring CCIs across multiple evaluation metrics, including prediction accuracy and the AUC values of ROC and PR curves. In addition, the proposed method exhibited competitive computational efficiency in terms of running time for CCIs inference.

We applied the proposed strategy to the COVID-19 severity-stage interaction. Our results show that COVID-19 genes that play key roles in the initial stages of COVID-19 continue to play crucial roles in the progression to severe stages. Specifically, FOS, CXCL8, HLA-A, JAK3, ICAM1, and H2BC4 were identified as high-severity-specific markers while IL1-β, CD3D, CD4, CCL5, and SNRPB were low-severity-specific markers. These results suggest that regulating the expression of high- and low-severity-specific markers may provide crucial clues to prevent progression to later stages of COVID-19. Additionally, the “*Systemic lupus erythematosus*” pathway appears to be significant in understanding the mechanisms underlying COVID-19 progression.

The proposed DD-CC-II offers several advantages. First, the strategy effectively infers cell–cell interactions (CCIs) by leveraging expression patterns without relying on predefined assumptions. Second, unlike many existing approaches, DD-CC-II operates independently of ligand–receptor databases, expanding its applicability to various biological contexts. Furthermore, the proposed method demonstrates competitive performance in terms of computational efficiency, despite the complexity of the task. We expect that the DD-CC-II could be a powerful tool for elucidating the cellular communication landscape in both healthy and diseased tissues, providing novel insights into immune regulation and disease progression at the CCI level.

DD-CC-II is a flexible, data-driven framework that can be applied to emerging technologies such as spatial transcriptomics and multi-omics data. In spatial transcriptomics, distinct tissue regions can be treated as “cell groups,” allowing DD-CC-II to infer cell–cell interactions within a spatial context and identify key interactions involved in disease progression. Moreover, its core principles—matrix decomposition (SVD) and correlation network construction—can be extended to other omics layers, such as proteomics or epigenomics, enabling the integration of multi-omics data for the comprehensive modeling of cellular communication and underlying biological mechanisms.

For estimating the eigen-cells in Monte Carlo simulation, we included a small portion of cells from other lineages to introduce realistic variability. Although using 0% of external cells is also possible, incorporating a minor fraction (1%, 3%, 5%, and 10%, etc.) better reflects potential stochastic effects across heterogeneous cell populations. The proportion of 5% was empirically chosen as a reasonable balance between stability and lineage specificity. Assessing the sensitivity of the method to different proportions will be an important direction for future work, as it may provide additional insights into the robustness and generalizability of the proposed approach.

To ensure sufficient sample size for robust statistical inference, we included only cancer types with at least two sublines containing at least ten cells each. This selection criterion ensures the reliability of our statistical analyses but may limit the generalizability of our strategy to less common cancer types. Therefore, caution should be exercised when extrapolating our strategy to rare cancers, as their cellular heterogeneity and interactions may not be fully captured in our current dataset.

## 4. Methods

The associations and dependencies between subjects were represented by a network of nodes connected by edges (links). Association strength was assessed by comparing the number of links. In this study, the network framework between the characteristics of the cell groups, as described by eigen-cells, was considered to infer CCIs. The interactions between groups were estimated using the eigen-cell correlation network. Finally, the significance of the associations between the groups was evaluated using over-representation analysis with the hypergeometric test.

### 4.1. Eigen-Cell Estimation

It was supposed that $X^{g}={\left(x_{1}^{g},\ldots ,x_{n_{g}}^{g}\right)}^{T}\in R^{n_{g}\times p}$ denotes the expression levels of *p* genes across $n_{g}$ cells for group *g*. Singular value decomposition (SVD) was applied for eigen-cell and/or eigen-gene analyses based on gene expression levels [51,52,53,54]. The SVD of $X^{g}\in R^{n_{g}\times p}$ was represented by

(1) $$\begin{array}{c} X^{g}=U^{g}D^{g}V^{gT}=\sum_{q=1}^{Q_{g}}d_{q}^{g}u_{q}^{g}v_{q}^{gT},\;g=1,\ldots ,G, \end{array}$$

where $U^{g}=\left[u_{1}^{g},\ldots ,u_{Q_{g}}^{g}\right]\in R^{n_{g}\times Q_{g}}$ and $V^{g}=\left[v_{1}^{g},\ldots ,v_{Q_{g}}^{g}\right]\in R^{p\times Q_{g}}$ are orthogonal matrices, and $D^{g}=\mathrm{diag}\left(d_{1}^{g},\ldots ,d_{Q_{g}}^{g}\right)$ with positive singular values $d_{1}^{g}\geq \ldots \geq d_{Q_{g}}^{g}$ on its diagonal. SVD is a linear transformation of gene expression levels $X^{g}$ from $n_{g}$ cell × *p* gene spaces to the reduced $Q_{g}$-eigen genes × $Q_{g}$-eigen-cells spaces, where $Q_{g}=\min\left\{n_{g},p\right\}$. The transformation matrices $U^{g}$ and $V^{g}$ represent the expression levels of $Q_{g}$-eigen genes in the $n_{g}$-cells and of *p*-genes in $Q_{g}$-eigen-cells [52]. Expression levels of *p* genes in $Q_{g}$ eigen-cells were considered; that is, $V^{g}=\left[v_{1}^{g},\ldots ,v_{Q_{g}}^{g}\right]$, as the characteristics of the *g*th group of cells and CCI inference was performed based on the estimated expression levels of eigen-cells. Details on the definition of eigen cells using SVD have been described elsewhere by Zou and Tibshirani [51].

### 4.2. Eigen-Cell Correlation Networks

A common strategy to measure the relationship between subjects is to consider the presence or absence of links connecting the nodes in the two subjects. We extended the co-expression networks of eigen-genes to the correlation network of eigen-cells to measure the association between groups of cells, that is, CCIs. The correlation between the *i*th eigen-cell of the *s*th group $v_{i}^{s}$ and the *j*th eigen-cell of the *t*th group $v_{j}^{t}$ were calculated using Pearson correlation coefficients [55,56]:

(2) $$\begin{array}{c} r_{ij}^{st}=\frac{\sum_{k=1}^{p}\left(v_{ki}^{s}-v_{-i}^{s}\right)\left(v_{kj}^{t}-v_{-j}^{t}\right)}{\sum_{k=1}^{p}{\left(v_{ki}^{s}-v_{-i}^{s}\right)}^{2}\sum_{k=1}^{p}{\left(v_{kj}^{t}-v_{-j}^{t}\right)}^{2}}, \end{array}$$

where $v_{ki}^{s}$ ($v_{kj}^{t}$) is the expression level of the *k*th gene in the *i*th (*j*th) eigen cell for group *s* (*t*); $v_{-i}^{s}$ and $v_{-j}^{t}$ are the averages of the *i*th and *j*th eigen-cells in groups *s* and *t*, respectively. The eigen-cell correlation network between groups *s* and *t* was constructed using significant eigen-cell pairs corresponding correlation coefficients with *p*-values below the threshold α (i.e., *p*-value ≤α). The strength of the association between cell groups was assessed by comparing the number of significant correlation coefficients with a reference distribution (i.e., over-representation analysis of eigen-cell pairs).

### 4.3. Cell–Cell Interaction Inferences

To measure the significance of the association between the cell groups (i.e., CCIs), an over-representation analysis was performed. That is, the strength of the association between groups was measured by the over-representation of the significant eigen-cell pairs.

For the query group of cells, *N* (*M*) represented the total number of all possible eigen-cell pairs (total number of eigen-cell pairs) between the query and all cell groups; *n* denoted all possible eigen-cell pairs between the query and target groups; *y* was a subset of *n* belonging to the target group. That is, *y* indicated the number of eigen-cell pairs (i.e., significant correlation coefficients) between the query and target cell groups. The observed *y* was considered the realization of a random variable *Y* that follows a hypergeometric distribution:

(3) $$\begin{array}{c} Y∼\mathrm{hypergeom}(n=n,K=M,N=N). \end{array}$$

The probability of over-representative eigen-cell pairs between the query and target groups was measured as

(4) P(Y=y)=MyN−Mn−yNn,

where ab is a binomial coefficient [57,58]. The significance of the over-representative eigen-cell pairs between the query and target groups was measured by the following hypergeometric distribution:

(5) p-value=1−∑i=0y−1MiN−Mn−iNn.

The association between the query and target cell groups was considered statistically significant when the FDR-q.value was below the significance level α; that is, the FDR-q.value ≤α, i.e., the Benjamini—Hochberg procedure for multiple testing correction was performed.

**Pipeline of DD-CC-II for CCIs**

1. SVD of expression levels of genes
  Given expression levels of genes for each group of cells, singular value decomposition is conducted to estimate expression levels of eigen-cells for groups.
2. Correlation coefficient network estimation
  We constructed correlation coefficient network of eigen-cells between groups based on significant eigen-cell pairs with the *p*.value ≤α.
3. Over-representation analysis of eigen-cell pairs
  Over-representation analysis of eigen-cell pairs is performed to measure the association between the groups of cells, where the significance of association was accessed by hyper geometric test with the FDR-q.value ≤α.

**Figure 6:**
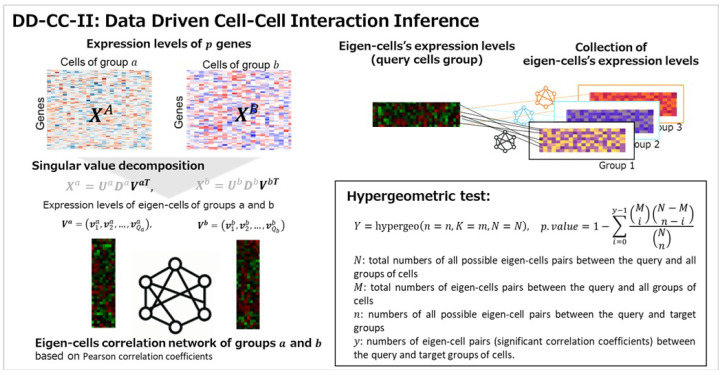
Overview of the data-driven cell–cell interaction inference. First, given the expression levels of genes for each cell group, singular value decomposition is conducted to estimate expression levels of eigen-cells for each group. Next, a correlation coefficient network for eigen-cells was constructed between groups based on significant eigen-cell pairs. Finally, the over-representation analysis of eigen-cell pairs is performed to measure the association between the cell groups, with association significance accessed using the hyper geometric test.

A schematic of the proposed data-driven cell–cell interaction inference is presented in Figure 6.

The SVD transformation in our framework serves as a biologically grounded approach to extract coherent functional modules from disease-relevant gene dysregulation, rather than a purely mathematical tool for dimensionality reduction. Specifically, differentially regulated genes are first identified using quantitative gene network meta-information to focus the analysis on functionally perturbed genes. Applying SVD to this filtered expression matrix decomposes the disease-related expression variability into orthogonal components, each representing a coordinated pattern of gene regulation corresponding to a functional module within the cell population. The resulting eigen-cells thus represent abstract functional circuits that summarize group-level biological behavior, rather than individual single-cell profiles. A significant correlation between an eigen-cell from the query and one from the target group reflects coordinated functional communication between these modules, providing a system-level interpretation of CCIs driven by network-level dysregulation.

## Figures and Tables

**Table 1:** Lineage subtypes of each cancer and cell numbers.

Lineage	Sub-Lineage	# Cells
Lung	NSCLC	135
	SCLC	50
	Mesothelioma	20
Blood	AML	44
	ALL	36
	CML	17
Lymphocyte	Non-hodgkin lymphoma	57
	Lymphoma unspecified	18
Breast	Breast ductal carcinoma	34
	Breast carcinoma	25
Soft tissue	Rhabdomyosarcoma	17
	Malignant rhabdoid tumor	12
Bone	Ewing sarcoma	21
	Osteosarcoma	16

**Table 2:** DepMap dataset: Numbers of eigen-cells (i.e., $Q_{g}$) for groups of cells, where “-” indicates that EGs-3 are unavailable and no eigen-cells were estimated for the group.

	EGs-1	EGs-2	EGs-3	EGs-n1	EGs-n2	EGs-n3
Lung	80.0	31.0	13.0	28.1	27.2	12.2
Blood	27.0	22.0	10.0	21.6	21.6	10.5
Lymphocyte	33.0	11.0	-	11.1	11.0	-
Breast	21.7	16.0	-	15.3	15.2	-
Soft tissue	10.0	9.0	-	8.8	8.8	-
Bone	13.8	10.0	-	9.9	10.0	-

**Table 3:** DepMap dataset: AUC values of ROC and PR curves in CCI inferences; values in parentheses indicate the standard deviation of AUC values across 50 simulation replicates.

		DD-CC-II		CellCall	CellChat	iTALK	REMI
		α=0.05	α=0.01				
ROC curve	Lung	**0.72**	0.70	0.20	0.51	0.44	0.63
		(0.15)	(0.14)	(0.16)	(0.09)	(0.16)	(0.15)
	Blood	**0.98**	0.97	0.21	0.44	0.12	0.53
		(0.04)	(0.00)	(0.18)	(0.16)	(0.12)	(0.10)
	Lymphocyte	**1.00**	**1.00**	0.63	0.10	0.16	0.75
		(0.00)	(0.00)	(0.28)	(0.13)	(0.17)	(0.22)
	Breast	0.97	**0.98**	0.21	0.48	0.40	0.26
		(0.16)	(0.09)	(0.24)	(0.34)	(0.29)	(0.22)
	Soft tissue	**0.73**	0.47	0.57	0.47	0.26	0.36
		(0.26)	(0.27)	(0.16)	(0.32)	(0.22)	(0.27)
	Bone	**0.95**	0.94	0.39	0.50	0.46	0.61
		(0.17)	(0.11)	(0.24)	(0.23)	(0.31)	(0.27)
PR curve	Lung	**0.89**	0.77	0.60	0.74	0.74	0.85
		(0.08)	(0.15)	(0.10)	(0.05)	(0.10)	(0.07)
	Blood	**0.99**	0.93	0.63	0.73	0.57	0.70
		(0.03)	(0.09)	(0.10)	(0.08)	(0.08)	(0.06)
	Lymphocyte	**1.00**	**1.00**	0.86	0.65	0.69	0.93
		(0.00)	(0.00)	(0.10)	(0.09)	(0.11)	(0.06)
	Breast	**0.99**	0.98	0.65	0.82	0.78	0.72
		(0.06)	(0.10)	(0.13)	(0.15)	(0.13)	(0.12)
	Soft tissue	**0.93**	0.80	0.85	0.84	0.77	0.77
		(0.09)	(0.11)	(0.05)	(0.13)	(0.12)	(0.14)
	Bone	**0.99**	0.97	0.81	0.86	0.82	0.89
		(0.04)	(0.17)	(0.14)	(0.08)	(0.12)	(0.10)

**Table 4:** Cancer types and number of cells in GDSC dataset.

GDSC Tissue Descriptor	Cancer Type	# Cells
Leukemia	ALL	24
	LAML	27
	LCML	10
Aero_dig_tract	HNSC	42
	ESCA	35
Digestive_system	LIHC	17
	STAD	27
Nervous_system	LGG	17
	GBM	35

**Table 5:** GDSC dataset: Number of eigen-cells (i.e., $Q_{g}$) for cell groups, where “-” indicates that EGs-3 are unavailable and no eigen-cells were estimated for the group.

	EGs-1	EGs-2	EGs-3	EGs-n1	EGs-n2	EGs-n3
LEUK	15	17	6	15.5	17.24	6.24
AERO	26	22	-	26.56	22.02	-
DIG	11	16	-	10.88	15.48	-
NERV	11	22	-	10.9	22	-

**Table 6:** GDSC dataset: AUC values of ROC and PR curves in CCI inferences; values in parentheses indicate the standard deviation of AUC values across 50 simulation replicates.

		DD-CC-II		CellCall	CellChat	iTALK	REMI
		α=0.05	α=0.01				
ROC curve	LEUK	**0.99**	0.98	0.27	0.07	0.14	0.43
		(0.03)	(0.05)	(0.15)	(0.09)	(0.06)	(0.11)
	AERO	**1.00**	**1.00**	0.19	**1.00**	0.96	0.32
		(0.00)	(0.00)	(0.22)	(0.00)	(0.11)	(0.31)
	DIG	**0.85**	0.78	0.13	0.40	0.53	0.44
		(0.21)	(0.23)	(0.20)	(0.23)	(0.08)	(0.33)
	NERV	**1.00**	**1.00**	0.01	**1.00**	**1.00**	0.83
		(0.00)	(0.00)	(0.04)	(0.00)	(0.00)	(0.19)
PR curve	LEUK	**1.00**	**0.97**	0.66	0.55	0.58	0.79
		(0.01)	(0.10)	(0.10)	(0.03)	0.04)	(0.07)
	(AERO	**1.00**	**1.00**	0.70	**1.00**	0.99	0.71
		(0.01)	(0.13)	(0.10)	(0.03)	(0.04)	(0.07)
	DIG	**0.96**	0.93	0.67	0.81	0.88	0.80
		(0.06)	(0.12)	(0.12)	(0.10)	(0.02)	(0.16)
	NERV	**1.00**	**1.00**	0.60	**1.00**	**1.00**	0.95
		(0.00)	(0.03)	(0.03)	(0.00)	(0.00)	(0.06)

**Table 7:** Execution time (in seconds) of CCI inference using DD-CC-II, CellCall, CellChat, iTALK, and REMI.

		DD-CC-II	CellCall	CellChat	iTALK	REMI
DepMap	Lung	0.75	2655.84	114.48	18.07	54.89
	Blood	0.76	2689.00	106.80	11.23	57.55
	Lymphocyte	0.69	2065.21	78.16	6.39	27.81
	Breast	0.97	1900.80	81.12	6.53	28.14
	Soft tissue	1.31	2021.19	76.14	4.72	28.93
	Bone	1.77	1927.36	76.45	4.96	27.72
GDSC	LEUK	1.25	1635.12	106.79	3.25	43.67
	AERO	0.60	923.76	60.47	3.61	20.82
	DIG	0.55	125.04	76.34	1.20	19.52
	NERV	0.60	310.05	74.12	2.66	18.53

**Table 8:** KEGG pathways of COVID-19 and immune disease.

Entry	Name	# Genes
hsa05171	Coronavirus disease-COVID-19	23
hsa05310	Asthma	32
hsa05322	Systemic lupus erythematosus	141
hsa05323	Rheumatoid arthritis	95
hsa05320	Autoimmune thyroid disease	54
hsa05321	Inflammatory bowel disease	66
hsa05330	Allograft rejection	39
hsa05332	Graft-versus-host disease	45
hsa05340	Primary immunodeficiency	38

**Table 9:** FDR-q.value for disease-trajectory correlations of COVID-19 stages.

	CCIs Inferences					
	Lv1–Lv2	Lv1–Lv3	Lv1–Lv4	Lv2–Lv3	Lv2–Lv4	Lv3–Lv4
COVID-19	0.000	0.000	0.000	0.187	0.002	0.583
Allograft rejection	0.458	0.606	0.102	0.512	0.211	0.125
Asthma	0.480	0.573	0.782	0.655	0.586	0.631
Autoimmune thyroid disease	0.384	0.526	0.251	0.156	0.286	0.297
Graft-versus-host disease	0.515	0.354	0.258	0.400	0.477	0.187
Inflammatory bowel disease	0.095	0.072	0.030	0.060	0.078	0.122
Primary immunodeficiency	0.471	0.015	0.038	0.080	0.034	0.697
Rheumatoid arthritis	0.977	0.002	0.011	0.001	0.045	0.211
Systemic lupus erythematosus	0.544	0.002	0.000	0.121	0.000	0.006

**Table 10:** Crucial genes in eigen-cell estimation for each COVID-19 severity stage.

Rank	Asymptomatic	Mild	Severe	Critical
1	HLA-B	HLA-B	HLA-B	HLA-B
2	RPS27	RPS27	NFKBIA	NFKBIA
3	RPL41	NFKBIA	HLA-C	HLA-C
4	NFKBIA	RPL41	RPS27	FOS
5	HLA-C	HLA-C	FOS	CXCL8
6	RPL13	CXCL8	CXCL8	RPS27
7	RPS29	RPS29	HLA-A	HLA-A
8	RPS11	FOS	RPL41	RPL41
9	RPS18	HLA-A	RPS11	HLA-E
10	RPL10	RPS11	RPS29	RPS11

**Table 11:** KEGG pathways associated with COVID-19 and immune disease.

	Rank	Asymptomatic	Mild	Severe	Critical
Inflammatory bowel disease	1	JUN	JUN	JUN	JUN
	2	TGFB1	TGFB1	TGFB1	TGFB1
	3	IL2RG	IL2RG	IL2RG	IL2RG
	4	RELA	STAT6	RELA	STAT6
	5	STAT6	RELA	STAT6	TLR2
	6	IFNGR2	IFNGR2	IFNGR2	RELA
	7	IL4R	IL4R	TLR2	IL4R
	8	IL1B	TLR2	IL4R	IFNGR2
	9	STAT3	IL1B	STAT3	STAT3
	10	TLR2	STAT3	STAT1	IFNGR1
Primary immunodeficiency	1	IL2RG	IL2RG	IL2RG	PTPRC
	2	PTPRC	PTPRC	TAP1	IL2RG
	3	TAP1	TAP1	PTPRC	TAP1
	4	CD3E	CD3E	CD3E	TAP2
	5	ZAP70	ZAP70	TAP2	JAK3
	6	CD79A	TAP2	ZAP70	RFXANK
	7	TAP2	CD3D	RFXANK	CD3E
	8	CD3D	RFXANK	JAK3	ZAP70
	9	CD4	CD4	CD3D	ORAI1
	10	RFXANK	CD79A	ORAI1	IKBKG
Rheumatoid arthritis	1	FOS	CXCL8	FOS	FOS
	2	CXCL8	FOS	CXCL8	CXCL8
	3	JUN	JUN	JUN	JUN
	4	ITGB2	ITGB2	ITGB2	ITGB2
	5	ATP6V0C	ATP6V0C	ATP6V0C	ATP6V0C
	6	TCIRG1	TCIRG1	TCIRG1	TCIRG1
	7	TGFB1	TGFB1	TGFB1	ATP6V0B
	8	ATP6V0B	CCL5	ATP6V0B	ICAM1
	9	CCL5	ATP6V0B	ICAM1	TGFB1
	10	LTB	ICAM1	CCL5	LTB
Systemic lupus erythematosus	1	FCGR3B	FCGR3B	FCGR3B	FCGR3B
	2	FCGR3A	FCGR3A	FCGR3A	FCGR2A
	3	FCGR2A	FCGR2A	FCGR2A	FCGR3A
	4	H2AC6	H2AC6	H2AC6	H2AC6
	5	SNRPB	MACROH2A1	MACROH2A1	H2BC12
	6	MACROH2A1	SNRPB	H2BC12	MACROH2A1
	7	H2AZ1	H2AZ1	H2BC4	H2BC4
	8	H2BC12	H2BC12	SNRPB	FCGR1A
	9	ACTN4	H2BC4	H2AZ1	H2AZ1
	10	ACTN1	H2AJ	ACTN1	ACTN1

## Data Availability

The datasets used in the Monte Carlo simulation section are from the DepMap database (https://depmap.org/portal/ (accessed on 1 February 2020)). The RNA-seq expression data of COVID-19 samples are available at the National Bioscience Database Center (NBDC) Human Database (accession code: hum0343; https://humandbs.biosciencedbc.jp/en/hum0343 (accessed on 1 April 2022)).
